# Research trends in lung cancer and the tumor microenvironment: a bibliometric analysis of studies published from 2014 to 2023

**DOI:** 10.3389/fonc.2024.1428018

**Published:** 2024-07-31

**Authors:** Zhilan Huang, Tingyi Xie, Wei Xie, Zhuni Chen, Zhiyuan Wen, Lin Yang

**Affiliations:** ^1^ The Fourth Clinical Medical College of Guangzhou University of Chinese Medicine, Shenzhen, Guangdong, China; ^2^ Department of Respiratory Medicine, Shenzhen Traditional Chinese Medicine Hospital, Shenzhen, China

**Keywords:** Lung cancer, the tumor microenvironment (TME), bibliometric, visualized analysis, trend

## Abstract

**Background:**

Lung cancer (LC) is one of the most common malignant tumors in the world and the leading cause of cancer-related deaths, which seriously threatens human life and health as well as brings a heavy burden to the society. In recent years, the tumor microenvironment (TME) has become an emerging research field and hotspot affecting tumor pathogenesis and therapeutic approaches. However, to date, there has been no bibliometric analysis of lung cancer and the tumor microenvironment from 2014 to 2023.This study aims to comprehensively summarize the current situation and development trends in the field from a bibliometric perspective.

**Methods:**

The publications about lung cancer and the tumor microenvironment from 2014 to 2023 were extracted from the Web of Science Core Collection (WoSCC). The Microsoft Excel, Origin, R-bibliometrix, CiteSpace, and VOSviewer software are comprehensively used to scientifically analyze the data.

**Results:**

Totally, 763 publications were identified in this study. A rapid increase in the number of publications was observed after 2018. More than 400 organizations published these publications in 36 countries or regions. China and the United States have significant influence in this field. Zhou, CC and Frontiers in Immunology are the most productive authors and journals respectively. Besides, the most frequently cited references were those on lung cancer pathogenesis, clinical trials, and treatment modalities. It suggests that novel lung cancer treatment models mainly based on the TME components, such as cancer-associated fibroblasts (CAFs) may lead to future research trends.

**Conclusions:**

The field of lung cancer and the tumor microenvironment research is still in the beginning stages. Gene expression, molecular pathways, therapeutic modalities, and novel detection technologies in this field have been widely studied by researchers. This is the first bibliometric study to comprehensively summarize the research trend and development regarding lung cancer and tumor microenvironment over the last decade. The result of our research provides the updated perspective for scholars to understand the key information and cutting-edge hotspots in this field, as well as to identify future research directions.

## Introduction

Lung cancer (LC) is a global health concern and one of the leading causes of cancer-related mortality. According to the global cancer statistics report published by the International Agency for Research on Cancer (IARC), incidence and mortality rates of lung cancer remain high, accounting for 18% of global cancer deaths in 2020 (1–3).

Surgery, radiotherapy, and chemotherapy have been the standard of care for lung cancer treatment in recent years. However, the clinical use of targeted therapies and immunotherapy has been increasing. The focus has shifted to detecting driver genes associated with tumor development, such as EGFR, KRAS, and MET, and identifying the signaling pathways of cell growth or apoptosis regulated by these genes. Targeting treatment to these genes has significantly improved the intermediate survival of lung cancer patients. Immunotherapy is now the standard first-line treatment for patients with advanced or metastatic mutation-negative driver genes in NSCLC. Unfortunately, tumor recurrence often leads to resistance to the initially effective drug (4).

With the emerging heated concept of tumor microenvironment (TME), increasing evidence suggests that TME promotes cancer progression and may mediate therapeutic resistance. Lung cancer-related therapies and studies are gradually expanding from focusing solely on the tumor cells themselves to the broader field of tumor microenvironment research. The development of cancer is strongly correlated with the physiological status of the tumor microenvironment, which can regulate tumor cells multiplication and bolster resistance to therapy. The TME is a hierarchically structured ecosystem that contains a variety of cell types ranging from tumor-associated macrophages (TAMs), immune cells, and cancer-associated fibroblasts (CAFs), as well as blood vessels, nerve fibers, extracellular matrix, and related noncellular components (5–7). In particular, immune cells play important roles in TME, which includes the promotion of tumor growth, and play a key role in host immune surveillance and elimination of neoplastic cancer cells (8). The cellular composition and functional status of the TME change depending on the tumor category, intrinsic characteristics of the cancer cells, tumor stage, and the characteristics of the individual patient. The effects of these cells can be mutual concerning the tumor and play a key role in host immunosurveillance and elimination of neoplastic cancer cells (9). Collectively, their interactions regulate regional immune effects and ultimately influence lung cancer outcomes, thus the cells in the TME and their secreted molecules are now considered to be critical in the pathogenesis of cancer for which they serve as potential targets for novel therapeutic cancer interventions.

TAM is also believed to be a key factor driving the TME to promote lung cancer development. It can be directly involved in tumor invasion, migration, epithelial-to-mesenchymal transition (EMT), and angiogenesis by secreting the chemokine CCL18. Ultimately, this leads to cancer progression and activates the NF-κB pathway in CAFs, inducing stemness and drug resistance in tumor cells (10, 11). The study by Xiang indicated that the increase in Tregs and dendritic cells (DCs) in the may also to acquired resistance after targeted therapy and immunotherapy (12). Based on scRNA-seq analysis and *in vitro* experiments, Aiko showed that high levels of IL-1β in TME may cooperate with IFN-γ to induce up-regulated expression of PD-L1 in tumor cells through activation of MAPK signaling, which in turn leads to resistance to tumor immunosuppression (13).

Besides, the TME can be subdivided into six specific categories: hypoxic ecological niche, immune microenvironment, metabolic microenvironment, acidic ecological niche, innervation ecological niche, and mechanical microenvironment (6). There is also bi-directional communication between microenvironments, so that targeting one specific microenvironment may result in a series of changes in other specific microenvironments and relevant pathways. As more and more studies have demonstrated the involvement of TME components in immune evasion and drug resistance against tumor cells (14–16). TAM is dynamic and subject to change due to pathogenic factors. Cigarette smoking is the most significant risk factor for lung cancer incidence and mortality (17, 18). Using a mouse cellular model, Bianchi discovered that exhaled tobacco smoke could induce the polarization of M2 phenotypic macrophages through various mechanisms. This ultimately hinders the anti-inflammatory effects of TAM in the TME of smokers with lung cancer, leading to the development of an immunosuppressive microenvironment (1). The study of lung cancer is shifting from a cancer-centered paradigm to one that considers the tumor microenvironment (TME) as a whole. It will be meaningful to monitor the dynamic pattern of development between changes in the TME and lung cancer.

Bibliometrics is an emerging method of literature analysis, which is a cross-discipline integrating mathematics, statistics, and bibliography. It is now widely applied by researchers, institutions, and countries in multiple disciplines and fields to build up all knowledge carriers into visual knowledge networks from quantitative and qualitative perspectives. Then through analyzing the big data intelligently the trend and current status of a particular research area can be derived, which will help to guide the policy decisions (19). Additionally, with bibliometric analysis, researchers can quickly, accurately, and comprehensively obtain detailed information, including the intellectual network, research topic evolution, potential development prospects, authors, collaborations, keywords, journals, countries, research institutes, references, and other details of the relevant research areas. Eventually, tools like CiteSpace, VoSviewer, R package, etc. can be used to visualize the results. Comparing it with the traditional literature review, the analysis based on bibliometrics provides a more comprehensive perspective of research trends with more objective data (20).

At present, there is no published bibliometric analysis available that covers lung cancer related to TME. Therefore, the research status as well as the research frontiers and hotspots in this field are still unclear. To provide a reference for further research and application, this paper recognizes and collects relevant literature data from databases. Literature analysis software has been used to study the annual number of publications, countries, publishing organizations, journals, authors, keywords, and references for the last 10 years at the intersection of TME and Lung Cancer, which will describe the progress, hotspots, and emerging trends of research in the field.

## Materials and methods

### Database and search strategies

**Figure 1:**
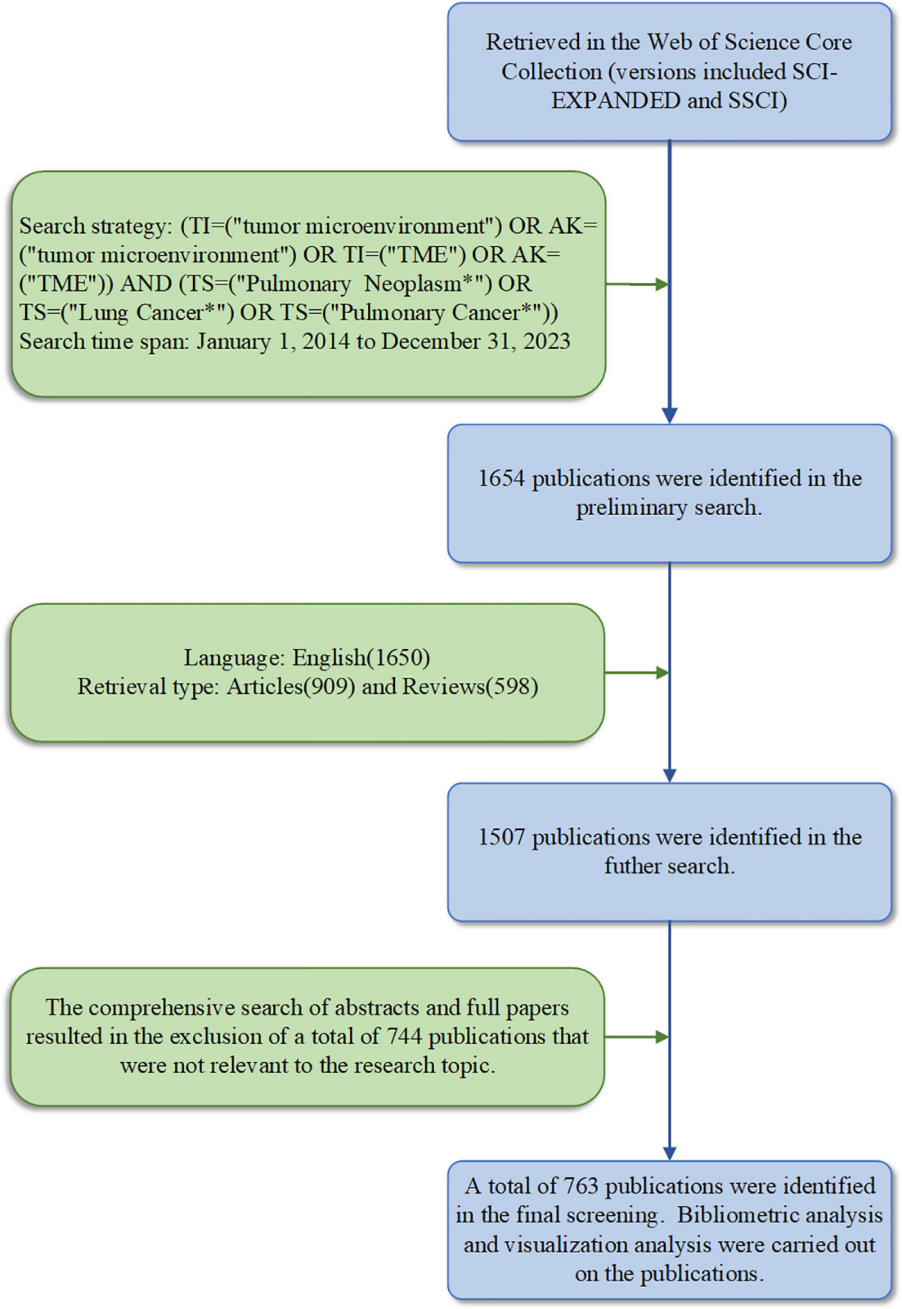
Process of publications selection in lung cancer and the tumor microenvironment.

The data source we used was the Web of Science Core Collection (http://wcs.webofknowledge.com), which includes the Science Citation Index Expanded (SCIE) and the Social Science Citation Index (SSCI). A comprehensive database search was completed by both authors on January 7, 2024. The specific search formula was as follows: (TI = (“tumor microenvironment”) OR AK = (“tumor microenvironment”) OR TI = (“TME”) OR AK = (“TME”)) AND (TS = (“Pulmonary Neoplasm*”) OR TS = (“Lung Cancer*”) OR TS = (“Pulmonary Cancer*”)). After the preliminary search, two authors (XTY and HZL) independently reviewed and screened the searched publications based on the following inclusion criteria (1): the publication timespan was set from 1 January 2014 to 31 December 2023 (2); only English-language publications were included (3); the publication type was limited to articles or reviews; and (4) the publication was related to a study of both lung cancer and the tumor microenvironment. In order to ensure the representativeness of the selected publications, the search results underwent a title and abstract-based filtration process, which excluded irrelevant publications. The screening criteria were shown in **Figure 1**. A total of 763 publications were included in the final analysis, which were eventually exported in “plain text” format with “full records and references”.

### Data analysis and visualization

Three scientometric software tools and two statistical mapping software were used in this study. Citespace (version 6.1.6, https://citespace.podia.com) is a Java-based information visualization and analysis software developed by Professor Chen, who specialized in Computer Science and Intelligence at Drexel University (21). After transforming the data using the software, we defined the analysis period as 2014–2023, the time interval as 1 year, the g-index as k = 25, and the node types as “author,” “institution,” and “keyword” for the co-occurrence network analysis. Each node represents a type of project. Plus, the size and color of the node circles indicate the number or frequency of publications and the year in which these projects appeared. The links between nodes reflect the collaboration between different projects. In addition, VOSviewer (version 1.6.19, https://www.vosviewer.com) (22) is a software tool designed by van Eck and Waltman, mainly used to construct and visualize metering networks. Visualization was achieved by analyzing the co-cited authors and co-cited publications through data importation. The bibliometrix package (version 4.2.2, http://www.bibliometrix.org) (23) in R can also play a role in analyzing and visualizing scientific literature. In our study, we primarily utilize a tool to count, analyze, and visualize various aspects such as national geographies, journal trends, top citations, and authors’ publications per year. Meanwhile, the number of publications per year and their respective countries are exported. Finally, the trends are predicted and visualized using Microsoft Excel 2019 and Origin 2021 software.

## Results

### Global trends of publication

**Figure 2:**
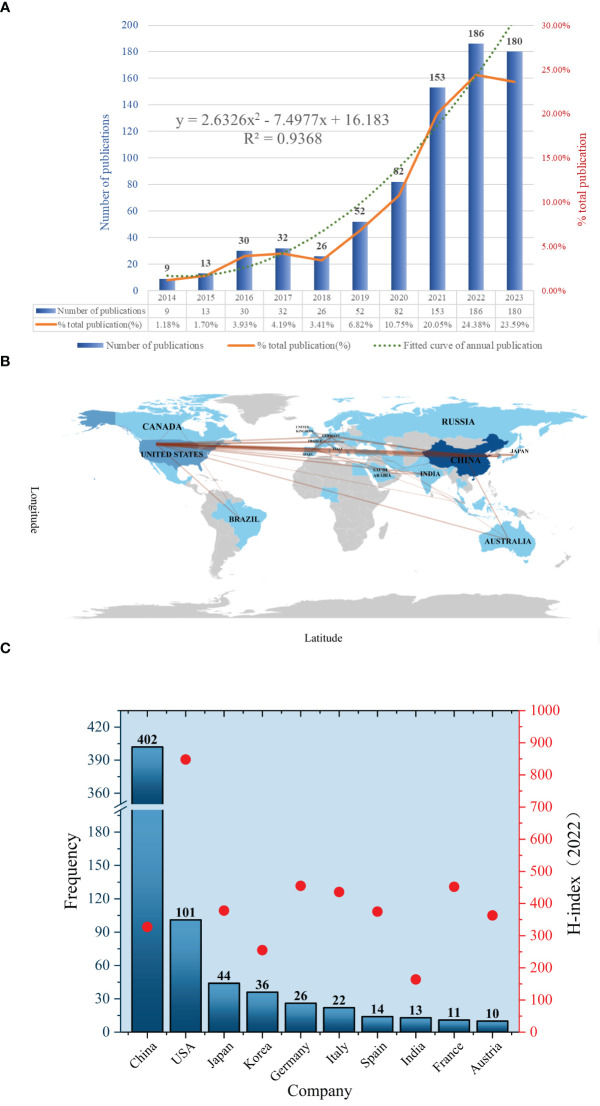
**(A)** Number of annual research publications and growth trends. **(B)** The visualization of country. **(C)** The geographical distribution.

The number of annual publications is an indicator of the developmental trend of scientific knowledge in a specific field. A total of 763 publications were selected to meet the inclusion criteria. This selection process is illustrated in **Figure 1**. The publications consisted of 174 reviews (22.8%) and 589 articles (77.2%), of which 4 non-English publications were excluded, and 143 publications were excluded due to inconsistent publications type. According to the analysis of publication numbers, the annual trend can be divided into two phases. From 2014 to 2018, the number of publications fluctuated between 9 and 32. However, the number of publications has shown an upward trend since 2019. It is shown that the number in 2022 surged to its highest level (186, 24.38%). The average annual number of publications was 76.3. Besides, the annual growth rate was 17.7%. A growth trend model was constructed using Microsoft Excel 2019 with the following equation: Y = 2.6326x^2^-7.4977x +16.183 (R² = 0.9368), where X represents the cumulative publication year (starting from 2014) and Y represents the annual publication (**Figure 2A**).

### Analysis of country or region

A total of 36 countries/regions have published relevant studies. The details of the top 10 most productive countries, along with their H-indexes in the field of oncology in 2022, are presented in **Figure 2B**. Upon reviewing the number of articles published by countries in this field in descending order, it is evident that China and the United States were the countries with the highest number of publications. In particular, the Chinese region ranked first, accounting for the greatest number of 402 articles (52.69%), followed by the United States with 101 (13.24%) and Japan with 44 (5.77%). The H-Index is a measure of a scholar’s or country’s level of scientific research. It is often used to assess the corresponding scholar’s or country’s influence and contributions to the academic field (24). By analyzing the H-index in the field of oncology, we found that the United States has the highest overall scientific impact on oncology research (score = 848), followed by the United Kingdom, Germany, and France. Although China is ranked first in the number of publications, its H-index is relatively low (score = 327), indicating a need to enhance both the quality and the academic impact of its research. As shown in the world map in **Figure 2C**, the overall distribution of publications within the country-region is clearly illustrated. The darker colors indicate a higher number of publications in those regions. It also illustrates the collaboration between countries and regions, such as China and the United States, China and Australia, and the United States and Brazil, etc. The single-country and multinational cooperative publications for the top 10 countries are presented in **Supplementary Table 1**. It can be observed that China (25) ranked first with the highest number of mutual co-publishing articles with other countries, followed by the United States (26) and Germany (9). However, considering the variation in the total number of publications per country, we utilize statistical analysis to compare them in terms of percentages. Consequently, the rate of cooperative papers published by Australia (70%) was much higher than that of France (54.5%), India (53.8%), and Germany (34.6%). Additionally, **Supplementary Table 2** presents the total citation data per country, with China ranking first (5,103), followed by the United States (3,284), and Japan (735). The average number of citations per paper for articles reflects the quality and influence of publications in each country. In sum, Australia ranks first in average citations with 94.8, while the United Kingdom comes in second with 93.

### Analysis of institutions

**Figure 3:**
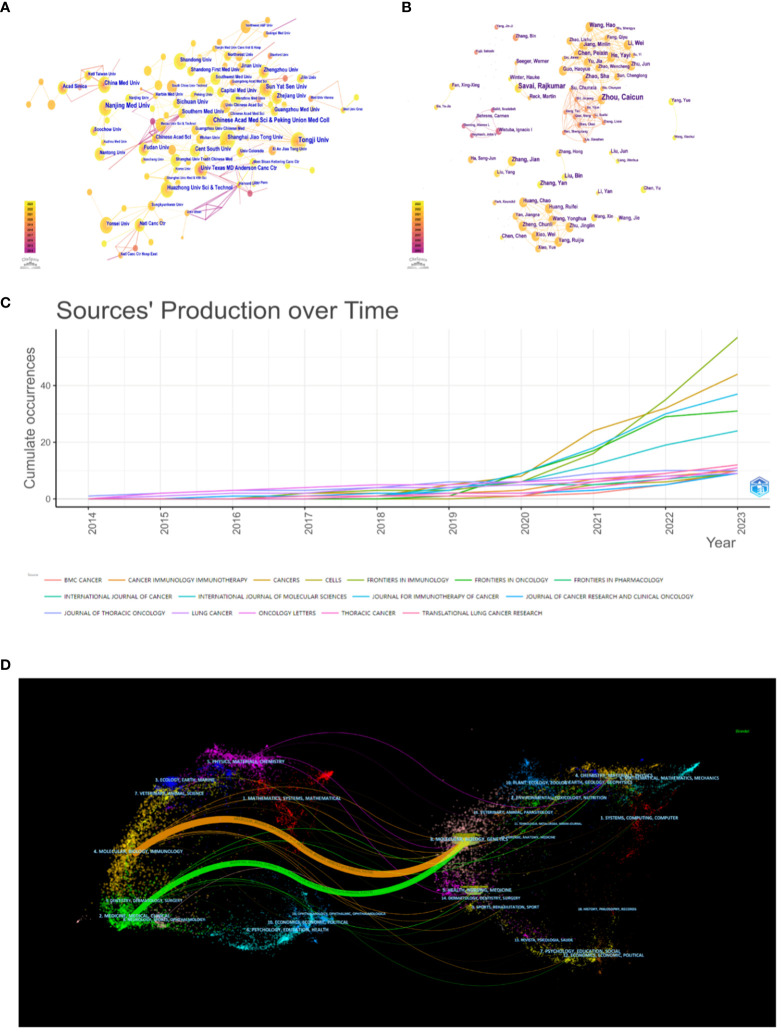
**(A)** The visualization of institution. **(B)** The visualization of author. **(C)** Cumulative publication trend of the top journals. **(D)** The dual-map overlay of journals.

More than 400 institutions engaged in research regarding xxx between 2014 and 2023. The top 10 most prolific institutes are shown in **Supplementary Table 3**. Meanwhile, we establish an institutional collaboration network by using the CiteSpace software (**Figure 3A**). Finally, it generates a total of 297 nodes and 477 edges, the numbers of which represent extensive cooperation between established institutions. The top 5 prolific institutions are Tongji University with 24 publications, Nanjing Medical University (21), Peking Union Medical College of the Chinese Academy of Medical Sciences (17), Sichuan University (16), and Sun Yat-sen University (16). What’s more, 20 institutions in total have published more than 10 publicaitons, with the majority of these research institutions located in China.

### Analysis of journals

Publications related to the tumor microenvironment and lung cancer have been published in 262 journals. The relative contents of the top 10 journals were listed in **Supplementary Table 4**, including information such as country, academic district, impact factor, H-index, and total citations. Frontiers in Immunology published the highest number of papers (n = 57, 7.47%), followed by Cancers (n = 44, 5.77%), Journal for Immunotherapy of Cancer (n = 37, 4.85%), and Frontiers in Oncology (n = 31, 4.06%). There are 11 journals with more than 10 publications. Impact factor (IF) and academic reputation are important criteria for evaluating research outcomes and academic excellence. The journal with the highest impact factor in 2022 is the Journal of Thoracic Oncology (IF = 20.4), followed by the Journal for Immunotherapy of Cancer (IF = 10.9) and Frontiers in Immunology (IF = 7.3). Although the majority of journals are primarily located in Europe, there are also a few in Asia, the Americas, and Oceania. The H-index of a journal usually refers to the number of journal publications that have been cited at least H times by other publications. It is also an indicator of academic quality and influence. In general, a journal with a higher H-index would obtain greater impact. The journal with the largest H-index and the largest total citations is the Journal for Immunotherapy of Cancer (H-index = 14, total citations = 877), while the journal with the second largest number of publications is Frontiers in Immunology (H-index = 13), Cancers (H-index = 11), and Frontiers in Oncology (H-index = 10). In addition to these journals, the top 3 cited journals also include the Journal of Thoracic Oncology (total citations = 732) and Frontiers in Immunology (total citations = 628). Visualization of the cumulative publications from the top 10 journals is shown in **Figure 3C** via the bibliometric package in R. A noticeable increasing trend in the publications of many journals can be observed. It is worth noting that Frontiers in Immunology and Cancers have demonstrated a particularly notable increase from 2020 to 2023, with growth exceeding that of other journals during the same period. The dual-mapped overlay of scholarly journals illustrates the relationship between citing and cited journals (**Figure 3D**). Labels indicate the subject areas of the journals, and colored lines represent different citation paths, with the width of the paths proportional to the z-score levels (27). The two main pathways were (1) molecular, biology, and immunology - molecular, biology, genetics (z = 5.8265, f = 5754) (2); medicine, medical, clinical - molecular, biology, genetics (z = 3.0848, f = 3173).

### Analysis of authors and cited authors

The top 10 most productive authors with the highest number of publications in this field are presented in **Supplementary Table 3**. Zhou, Caicun from Tongji University Affiliated Shanghai Pulmonary Hospital, and Savai, Rajkumar from the Department of Lung Development and Remodeling were the two most prolific authors, with 10 and 8 publications, respectively. Moreover, most of them are from China, which suggests that China is still in the leading position in this field. According to the author cooperation interrelationships in this field drawn by citespace (**Figure 3B**), a total of 382 nodes and 794 edges, indicate that authors with a higher number of papers typically collaborate with regular co-authors and teams.

Co-cited author analysis is a method used to assess the influence and contributions of authors within the academic community based on how frequently they have been cited in scholarly literature. This type of analysis can help researchers gain insights into academic trends, research hotspots, and academic authorities in a particular field.

**Figure 4:**
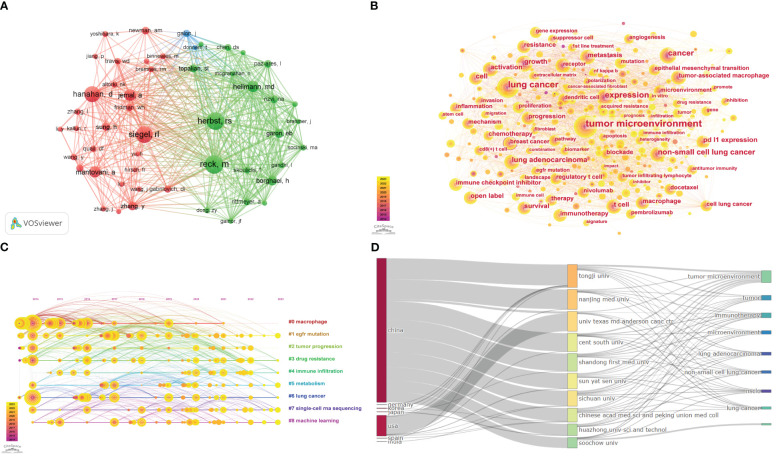
**(A)** The co-cited authors analysis. **(B)** The visualization of keyword. **(C)** Timeline view for keywords. **(D)** The three-field plot of lung cancer and the tumor microenvironment.

The network structure of co-cited authors was analyzed using VOSviewer software, as depicted in **Figure 4A**. The analysis revealed a total of 26,096 co-cited authors, with 51 authors cited more than 40 times. It can be observed in **Supplementary Table 5** lists the top 10 cited authors, their affiliations, and the H-index. Martin Reck from the LungenClinic Grosshansdorf had the largest total number of citations (n = 178), followed by Herbst, Roy S. from Yale University (n = 172) and Rebecca L. Siegel from the American Cancer Society (n = 161). Most of these co-cited authors are from Europe or North America. Furthermore, the author with the highest H-index among the top 10 is Alberto Mantovani (187), who has made a significant impact on scholarship in this field. Following Mantovani is Ahmedin Jemal (139) in second place, and then Douglas Hanahan (109).

### Analysis of keywords and timeline

The keyword co-occurrence analysis involves extracting and analyzing the keywords in the publication. Through the above process, it can help us identify the main topics, core contents, and key points so that the information can be well understood and utilized (26). The timeline view map is typically used to illustrate the evolution of specific research subjects or keywords over time, which enables researchers to observe research hotspots, citation relationships, and trends related to a particular topic or field across distinct timeframes. Furthermore, it facilitates an enhanced understanding of research dynamics and field evolution. The visualization of the relationship between keywords (**Figure 4B**) is achieved by constructing a visual network using the Citespace software, which consists of 401 nodes and 2766 edges. The size of the node is proportional to the frequency of occurrence of the associated keywords within the field. The larger the node, the greater the frequency of occurrence. Contour color reflects the year of occurrence, with darker colors indicating earlier years, while centrality indicates key nodes within a network. The top 20 keywords and their centrality are shown in **Supplementary Table 6**. Only the keyword “breast cancer” has a centrality greater than 0.1, while the rest have centrality values below 0.1. This indicates that there are few key nodes in the network. Keywords with higher frequencies include “tumor microenvironment,” “lung cancer,” “expression,” “cancer,” “non-small cell cancer,” and so on. As demonstrated in **Figure 4C**, the evolution of the tumor microenvironment and lung cancer in this field is delineated through the log-likelihood ratio (LLR) algorithm, the modularity value (Q-value), and the mean silhouette (S-value), which serve as crucial metrics for evaluating the outcomes of graph plotting. The Q-value of the graph, which reached 0.3555 (>0.3), was rationalized into loosely coupled clusters, and the homogeneity within the clusters was credible. The S-value of 0.6853 indicates that the clustering configuration is reasonable and is divided into nine clusters. These clusters are based on the keywords “macrophage,” “EGFR mutation,” “tumor progression,” “drug resistance,” “immune infiltration,” “metabolism,” “lung cancer,” “single-cell RNA sequencing,” and “machine learning.” Additionally, the association between the top 10 countries, institutions, and shared keywords was analyzed using R-bibliometrix (**Figure 4D**).

### Analysis of co-citation and burst reference

A total of 763 publications have been cited 13,663 times, with an average of 17.91 citations per paper. The top 10 most frequently cited publications are presented in **Supplementary Table 7** (28–37). The article by Koyama S et al. (32), published in Cancer Research in 2016 and titled “STK11/LKB1 Deficiency Promotes Neutrophil Recruitment and Proinflammatory Cytokine Production to Suppress T-cell Activity in the Lung Tumor Microenvironment,” ranked first in this field with a total of 374 citations. Three reviews were included in the top 10 most cited publications. The most cited article was published by Koyama S et al. (32), which received an average of 41.56 citations per year, followed by Bremnes Rm et al. (29), whose article was cited 37.11 times per year.

**Figure 5:**
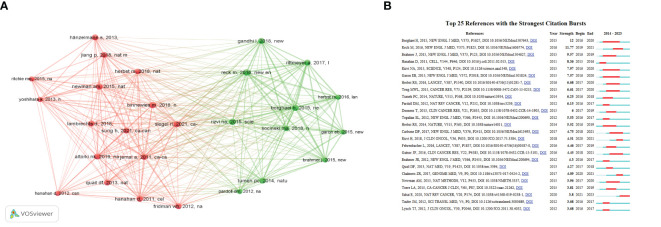
**(A)**The co-cited references analysis. **(B)** The top twenty-five references with the strongest citation bursts.

In the field, there are 36,868 co-citations available, out of which 72 references had more than 20 co-citations. As demonstrated in **Figure 5A**, the relational network graph, comprising more than 35 co-citations selected through the VOSviewer software, has a total of 322 edges and a total link strength of 1882. Besides, the top 10 co-cited references are listed in **Supplementary Table 8** (25, 38–46). The reference “Global Cancer Statistics” by Ahmedin Jemal in CA: A Cancer Journal for Clinicians had the greatest number of citations (25), while “Hallmarks of Cancer: The Next Generation” (40) and “Cancer Statistics, 2021” (45) follow closely. As can be seen in the table, the top ten co-cited references were mainly published between 2011 and 2021. With two articles published in the New England Journal, two in CA: A Cancer Journal for Clinicians, and the remaining articles published in various journals.

An outbreak of co-citation refers to literature that has been cited together by a wide range of researchers within a specific period. Based on a co-citation literature analysis of 763 documents imported into Citespace, the blue timeline in the figure illustrates the strongest citation bursts. These bursts are defined as the periods between co-cited references by different researchers. The red segments on the timeline represent the time intervals between bursts and indicate the start and end years of the bursts (47). The top 25 references with the strongest citation bursts are displayed in **Figure 5B**, as identified by the Citespace software. These bursts occurred as early as 2015 and as late as 2023. The most significant citation bursts were published in 2015 by Borghaei et al. (39) in the New England Journal, with a burst intensity of 12 and an outbreak period from 2016 to 2020. Overall, the citation burst intensity of these 25 references ranged from 12 to 3.68. The majority of the strongest citation bursts were published in the New England Journal of Medicine (7).

## Discussion

As far as incomplete statistics are concerned, this paper is the first bibliometric article in this field. The study involves a statistical analysis of the hotspots and trends related to lung cancer and the tumor microenvironment over the past decade using bibliometrics. This analysis utilizes software tools like Citespace, VOSviewer, and the R programming language. As illustrated in **Figure 2A**, the research in this field demonstrates a persistent upward trend, with a non-significant growth rate observed between 2014 and 2018. However, the number of publications has exhibited a notable acceleration since 2019, reaching levels comparable to those observed in 2022 and 2023. The number of annual publications has surpassed 100 for the first time in 2021, suggesting that this field is gradually attracting widespread attention from researchers, and the prospect of development is promising. The number of annual publications in the field is expected to reach approximately 650 by 2030, as predicted in the fitted model. By analyzing national and regional publications, it is evident that China and the United States are the primary research hubs in this field. This trend is closely linked to the concentration of research technology and top-tier talent in both countries. In addition, another important factor contributing to the annual increase in the number of articles published in China is likely associated with the large population base in China and the continuously rising incidence and mortality rate of lung cancer (48), a topic that receives widespread attention and support for research. The data presented in **Supplementary Table 1** indicate a relatively low proportion of collaborative research among countries in this field, with the majority of such research mainly being conducted in Europe and Oceania. Moreover, the average citation ranking of articles suggests that academic research conducted through international collaborations is more likely to attract greater attention and have a higher academic impact. While the East Asia region shows a significant publication volume, the level of inter-country cooperation is comparatively low. Therefore, it is evident that cooperation across countries will contribute to improving resource efficiency and academic development in this field in the future.

Research institutes refer to organizations or units specialized in scientific research, technological development, and innovation. This typically includes universities, research institutes, laboratories, and similar entities. These institutions can reflect the background and sources of research findings, as well as directly affect the quality and credibility of academic research. This study finds that the primary research institutions in this field are situated in China, which is closely associated with the large number of publications in the Chinese region. Meanwhile, Tongji University in China, which ranked first, has been at the forefront of lung cancer research, strongly correlated with the institution’s investment and adequate resources in research. As shown in **Supplementary Table 4**, most journals published in this field are predominantly located in the Q1 and Q2 quartiles of the Journal Citation Reports (JCR), indicating that they are highly valued by researchers worldwide. Nevertheless, the impact factor of the journals is relatively low, suggesting that scholarship in this area of research still needs improvement.

In terms of authors, Prof. Caicun Zhou from Tongji University in China is the most productive author. He has a long-term involvement in research on the molecular mechanisms and clinical effectiveness of drugs in lung cancer. In particular, Prof. Zhou has made significant contributions in the following areas: mechanisms of lung cancer drugs, immunotherapy for lung cancer, and the establishment of a model for predicting the risk of recurrence of lung cancer (37, 49–51). The following most prolific author is Professor Rajkumar Savai from the Department of Lung Development and Remodeling. He specializes in researching the association between lung cancer and the tumor microenvironment through tumor signaling pathways, macrophages, immunotherapy, etc. (52–55).

The most cited article in this field is a paper published in 2016 by Koyama S et al. regarding the effect of STK11/LKB1 deletion on the immune microenvironment in a KRAS-driven mouse model of NSCLC. The study found that immune escape is mediated by the suppression of myeloid cells and aberrant cytokine production in LKB1-deficient tumors (32). Next is a review of tumor-infiltrating lymphocytes (TILs) and non-small cell lung cancer by Bremnes Rm and colleagues in 2016 (29), as well as a review of the tumor microenvironment and metastatic mechanisms in lung cancer by Wood Sl et al. in 2014 (35).

### Research hotspots and frontiers

Keywords and cluster analysis are significant tools for identifying the hotspots and frontiers of research. As a result, we summarize the analysis to provide insight into the interconnection between lung cancer and the tumor microenvironment, suggesting several potential directions for future research.

The first topic to be considered is the relationship between gene expression and signaling pathways in lung cancer in the context of the tumor microenvironment. From the clustering in **Figure 4C**, it can be concluded that macrophages represent a significant category within the field of study. These cells commonly originate from bone marrow hematopoietic stem cells and subsequently differentiate into circulating monocytes in the peripheral blood. Then they migrate to various tissues and organs within the body, including the skeletal system, lungs, and liver. In these locations, they eventually transform into macrophages with specificity (56, 57). Specifically, macrophages can be divided into two subtypes depending on their activation status, function, and secreted cytokines. One subtype, M1-type macrophages, is activated by LPS, IFN-γ, or TNF and secretes high levels of pro-inflammatory cytokines. These cytokines play a role in the cellular immune response facilitated by type I helper T cells. The other subtype, M2-type macrophages, is polarized by Th2 cytokines. They induce immunosuppression, participate in pro-carcinogenic functions, and promote tumor growth and metastasis (58–60). Liu et al. (61) demonstrated that glucose metabolic pathways are intricately linked with polarization state shifts in tumor-associated macrophages. M1 macrophages upregulate glycolysis and the pentose phosphate pathway to trigger inflammation, while M2 macrophages rely more on the tricarboxylic acid cycle and mitochondrial metabolism to suppress anti-tumor immunity and promote tumor metastasis. In addition, tumor-associated fibroblasts (CAFs) were identified as a potential trend and direction for future research in this field, based on the outbreak of cited literature. Cancer-associated fibroblasts (CAFs) influence the creation of extracellular matrix (ECM) structures and metabolism in the TME. They play a crucial role in regulating tumor immunity and resistance to chemotherapeutic agents (62). Qiao et al. (63) found that in KRAS-mutant lung adenocarcinoma, STK11/LKB1 mutation by constructing a mouse model of lung cancer. Adhesion plaque kinase (FAK) inhibitors inhibited the activation of CAFs and further promoted the infiltration of CD8 T cells, DC cells, and M1-type macrophages into the tumors, thus remodeling the tumor microenvironment. Samart et al. (64) concluded that Musashi-2 (MSI2) has a potential impact on CAFs in regulating the invasive and metastatic spread of NSCLC cells by analyzing genomics and proteomics data. Additionally, a new complex regulatory axis involving MSI2/IL-6 was identified, indicating an interaction between NSCLC-derived CAFs and NSCLC cells through paracrine signaling. Cords et al. (65) found differences in the spatial distribution of distinct CAF phenotypes in TME and identified specific CAF phenotypes that were associated with good versus poor patient prognosis. Tumor metabolism essentially refers to the abnormal metabolic features of tumor cells that contribute to the proliferation and progression of tumors. This involves glucose uptake and utilization, as well as nucleotide synthesis. While metabolism-targeted therapies are widely discussed nowadays (66, 67). In line with the research focus of this field, lung cancer offers a promising path for exploring tumor metabolism and tumor microenvironment in precision medicine. Tumor metabolism has a significant impact on the tumor microenvironment, thus affecting the proliferation of lung cancer cells. Moreover, this research area offers insights that can inform the treatment of lung cancer (68). Liu et al. (69) predicted the prognosis of lung adenocarcinoma (LUAD) and the efficacy of various immunotherapies by constructing a model of glutamine metabolism. Furthermore, a research has demonstrated that deoxypodophyllotoxin (DPT) inhibits glycolysis by preventing the overexpression of HIF-1α, which in turn suppresses cell proliferation in NSCLC (70).

Secondly, we will be examining the links and interactions between lung cancer treatment and the tumor microenvironment. The analysis of keywords and clusters has revealed that scholars have devoted considerable attention to the treatment of lung cancer research, including studies on tumor progression and drug resistance. Firstly, the conventional treatment options include surgical intervention, radiation therapy, and chemotherapy. Surgery is considered the most effective clinical treatment for lung cancer (71). Yet, surgery is often limited to patients with stage I and II operable NSCLC and a preferred local treatment modality is recommended (8, 72). On the other hand, radiotherapy applies to all stages of lung cancer, affecting the functions of the immune system in various ways. With advances in imaging, radiotherapy is now faster and more precise in the treatment of lung cancer (73). Chemotherapy has a high frequency of keyword occurrences in this field, and platinum-based adjuvant chemotherapy is commonly used in patients diagnosed with stage II and stage III NSCLC (74). A combination of two cytotoxic drugs is recommended as first-line treatment for advanced metastatic NSCLC (8). However, the chemotherapy drugs can cause central and peripheral neurotoxicity, cardiotoxicity, gastrointestinal toxicity, and hematologic toxicity in humans (75, 76). From the perspective of immune mechanisms, chemotherapeutic agents can enhance the immune response by inducing immunogenic cell death, potentiating T-cell activation, and increasing the activity of tumor-killing immune cells (77). Whereas, the tumor microenvironment also contributes to an increase in resistance to chemotherapy drugs (78). With ongoing research on tumor molecules, targeted therapy is becoming a crucial treatment for non-small cell lung cancer. EGFR, KRAS, and ALK are the primary susceptibility genes for common NSCLC driver mutations (79, 80). Among these, EGFR mutation is a crucial factor for cluster analysis in this field. Targeted therapies exert their effects primarily on tumor cells by blocking specific signaling pathways. In addition, targeted therapies have a direct effect on tumor cells mainly by blocking specific signaling pathways, which the immune microenvironment interacts with to affect targeted drug sensitivity (81).

The tumor microenvironment also influences targeted drug resistance. EGFR mutations may decrease the number of CD8+ cells, elevate the Treg population, and activate the STAT-3 intracellular pathway, leading to immune escape and increased resistance to targeted drugs (82). Moreover, vascular endothelial growth factor (VEGF)-targeted drugs can affect the tumor microenvironment in NSCLC by inhibiting immune escape, normalizing tumor vasculature, modulating T-cell numbers, and increasing tumor immune cells (83). The field of immunotherapy has seen significant progress in the last decade, introducing new therapies that have become a standard treatment for patients with stage III or IV NSCLC. The tumor microenvironment plays a significant role in determining both the sensitivity and resistance to immune drugs (84). Immune checkpoint inhibitors (ICIs) represent another significant cluster in this field, encompassing a range of molecules, including PD-1, PD-L1, and CTLA-4, which exert anti-tumor effects by regulating the interaction of Treg cells with antigen-presenting cells or tumor cells (85). The antitumor efficacy of PD-1/PD-L1 blockers has been shown to correlate with an increased presence of CD8+ tumor-infiltrating lymphocytes and the overexpression of chemokines and cytokines in the tumor microenvironment (86). The efficacy of immune checkpoint inhibitors is linked to the activation of effector immune cells, such as tumor-infiltrating lymphocytes, dendritic cells, and others. Conversely, resistance is mainly associated with the infiltration of immune cells, including regulatory Treg cells, myeloid-derived suppressor cells (MDSCs), and tumor-associated macrophages, as well as the recruitment of chemokines and high expression of vascular endothelial growth factor (VEGF) (87). Compared with NSCLC, SCLC has no significant effect on immunotherapy. In addition to the low expression of PD-L1 in tumor cells, various factors such as low expression or deletion of MHC I and MHC II proteins in the tumor microenvironment, and inhibition of the proliferation of CD4+ cells can also contribute to immune escape (88).

Thirdly, the discussion will focus on the impact of new technologies and methods on the direction of research within this field. Single-cell RNA sequencing (scRNA-seq), one of the crucial research techniques in the field, is a novel approach that allows for the examination of the transcriptome of individual cells within a sequenced sample. This method facilitates the analysis of cell types and heterogeneity in gene expression (89). Hu et al. (90) demonstrated, using single-cell techniques and *in vivo* experiments, that tumor-associated macrophages (TAMs) promote IL-6 expression through the formation of an IL6-STAT3-C/EBPβ-IL6 positive feedback loop. This loop, in turn, induces the epithelial-to-mesenchymal transition (EMT) pathway as a mechanism to enhance migration, invasion, and metastasis in lung cancer. Han (91) found that Osimertinib, in combination with anti-angiogenic agents, increased the number of CD8 T cells and proliferation of T cells compared with a single agent by analyzing tumor tissue using ScRNA-seq. Mao (92) demonstrated that the expression of the CDC25C gene affects the invasion and migration of lung cancer cells through the study and analysis of scRNA-seq data, suggesting it may play a crucial role in the EMT pathway. Machine learning is also a significant research area in this field. It primarily focuses on the general concept of various models and strategies. Currently, machine learning methods are experiencing a gradual increase in the field of medical research (93). Cury et al. (94) employed machine learning modeling to predict the impact of the pectoralis major muscle region on NSCLC.

### Limitations

This study is subject to certain limitations. Firstly, the research data were exclusively retrieved from the SSCI and SCI-E databases of WoSCC, which may lead to incomplete data and related results. Secondly, the data we selected were exclusively published in English. This exclusion of books, conference papers, and other types of publications may have resulted in the omission of some articles. Thirdly, although this study employed rigorous screening criteria and a comprehensive double search and review process, the search formula may not fully encompass all relevant research findings in this field, potentially leading to the omission of crucial research contributions. Fourthly, since the software is not analyzed in the same manner, errors may exist in some of the results.

## Conclusions

In this research, bibliometric methods were used to visualize articles on lung cancer and the tumor microenvironment published between 2014 and 2023. This approach enabled researchers to gain into the current status, frontiers, and hotspots in this field. The findings indicate that the number of publications in this field is generally increasing. The majority of these publications are authored by researchers in China and the United States. Additionally, research on the correlation between the tumor microenvironment and lung cancer molecular signaling pathways and therapy is gaining increasing attention. These fields are expected to be significant focal points for future research on lung cancer and tumor microenvironment.

## Data availability statement

The original contributions presented in the study are included in the article/**Supplementary Material**. Further inquiries can be directed to the corresponding author.

## Author contributions

ZH: Data curation, Formal Analysis, Investigation, Methodology, Project administration, Supervision, Validation, Visualization, Writing – original draft, Writing – review & editing. TX: Formal Analysis, Investigation, Software, Supervision, Validation, Visualization, Writing – original draft, Writing – review & editing. WX: Funding acquisition, Project administration, Resources, Supervision, Validation, Writing – original draft, Writing – review & editing. ZC: Data curation, Investigation, Software, Validation, Visualization, Writing – review & editing. ZW: Investigation, Methodology, Software, Validation, Visualization, Writing – review & editing. LY: Investigation, Methodology, Software, Validation, Visualization, Writing – review & editing.
